# Microsecond time-scale kinetics of transient biochemical reactions

**DOI:** 10.1371/journal.pone.0185888

**Published:** 2017-10-03

**Authors:** Sandra Mitić, Marc J. F. Strampraad, Wilfred R. Hagen, Simon de Vries

**Affiliations:** Department of Biotechnology, Delft University of Technology, Delft, The Netherlands; Russian Academy of Medical Sciences, RUSSIAN FEDERATION

## Abstract

To afford mechanistic studies in enzyme kinetics and protein folding in the microsecond time domain we have developed a continuous-flow microsecond time-scale mixing instrument with an unprecedented dead-time of 3.8 ± 0.3 μs. The instrument employs a micro-mixer with a mixing time of 2.7 μs integrated with a 30 mm long flow-cell of 109 μm optical path length constructed from two parallel sheets of silver foil; it produces ultraviolet-visible spectra that are linear in absorbance up to 3.5 with a spectral resolution of 0.4 nm. Each spectrum corresponds to a different reaction time determined by the distance from the mixer outlet, and by the fluid flow rate. The reaction progress is monitored in steps of 0.35 μs for a total duration of ~600 μs. As a proof of principle the instrument was used to study spontaneous protein refolding of pH-denatured cytochrome *c*. Three folding intermediates were determined: after a novel, extremely rapid initial phase with τ = 4.7 μs, presumably reflecting histidine re-binding to the iron, refolding proceeds with time constants of 83 μs and 345 μs to a coordinatively saturated low-spin iron form in quasi steady state. The time-resolution specifications of our spectrometer for the first time open up the general possibility for comparison of real data and molecular dynamics calculations of biomacromolecules on overlapping time scales.

## Introduction

To study catalytic mechanisms of biochemical reactions, the mixing of two, or more, reactants must be sufficiently fast to cover the pre-steady state time domain in which the formation and decay of all reaction intermediates can be monitored. Rapid mixing is the most generally applicable method for reaction initiation. The pre-steady kinetics of enzymes can be monitored by a variety of spectroscopic techniques employing turbulent/laminar continuous-flow or stopped-flow rapid mixing instrumentation, as well as rapid freeze-quenching/sampling methodologies [1].

Continuous flow is at present the fastest option available, however, the dead-time of thus far described mixing equipment is still too slow (at least 45–50 μs [2–4]) to study the very onset of catalysis by many enzymes and the early folding events of proteins. In our design of an instrument capable of mixing reactants and observing the reaction progress on the microsecond time scale both the mixer and the optical observation cell had to be miniaturized to a typical dimensional scale of circa 100 μm, while concomitantly high mixing efficiency and satisfactory optical quality had to be maintained to enable high-resolution pre-steady-state kinetic analyses.

We have build a spectrometer that comprises a continuous flow microsecond time-scale mixing device tightly fitted to a high-pressure optical transmission cuvette. Equipped with a xenon lamp plus high-speed scanning monochromator as the light source, and with a CCD camera as the detector, the computerized setup provides unprecedented time resolution and excellent sensitivity as shown in very rapid acid-base and electron-transfer test reactions.

To illustrate its applicability and versatility we have used the instrument to measure very rapid phases in the spontaneous re-folding of acid-unfolded equine heart cytochrome *c* with subsequent identification of intermediates by numerical analysis with singular value decomposition. This study revealed a hitherto unidentified very early folding intermediate, which we identify as the histidine-18 re-coordinating to the iron(III) metal by replacement of a water ligand. The study also shows a possible practical limitation in terms of a minimal requirement of sensitivity for optical absorption as determined by the product of soluble-protein concentration and extinction coefficient(s).

## Materials and methods

### Chemicals

HPTS (8-hydroxypyrene-1,3,6-trisulfonic acid, or pyranine) was supplied by Invitrogen, Molecular Probes. Cytochrome *c* from equine heart, sodium ferrihexacyanide, and ascorbic acid were from Sigma Aldrich.

### Stopped-flow experiments

Conventional stopped-flow experiments were performed with an Applied Photophysics SX21 instrument for which the dead-time was determined at 2.5–2.6 ms. Experiments were performed in the stopped-flow photo-diode array mode, allowing absorbance spectral recording in the wavelength range from 195 nm to 740 nm. In all experiments, 29.6 μM ferrocytochrome *c* (reduced by 60 μM of ascorbic acid) was mixed with sodium ferrihexacyanide at concentrations varying from 1–20 μM. Kinetic measurements were performed in 50 mM phosphate buffer at pH 7, at two different temperatures, 19.3°C and 34.4°C. Acquired kinetic absorbance spectra were analyzed by either using the whole spectrum and applying two-component analysis (below) or by extracting the single wavelength traces at 550 nm.

### Continuous-flow experiments

Continuous-flow kinetic measurements with the new setup were performed at a total flow rate of 20 mL min^-1^ at 27.5°C in the spectral range from 350 nm to 600–650 nm. Data were collected as multi-scans, in general in steps of 1 nm per scan, each scan containing 2048 pixel time points. For dead-time determination with a fast electron-transfer reaction a stock solution of 2.2 mM ferrocytochrome *c* (the oxidized form reduced by 4.4 mM of ascorbic acid) was diluted to 0.96 mM; this latter solution was mixed with different sodium ferrihexacyanide concentrations varying from 1–100 mM. All solutions contained 50 mM phosphate buffer pH 7.0. In separate experiments oxidized or reduced cytochrome *c* (0.96 mM) were mixed versus buffer only. The data from these latter experiments served as the basis set for two-component analysis. Optical reference solutions contained bovine serum albumine (20 mg mL^-1^) in 50 mM phosphate buffer pH 7.0; these reference solutions were mixed with buffers containing the same ferrihexacyanide concentrations as in the oxidation experiments.

Refolding experiments of acid denatured cytochrome *c* were performed at flow rates of 20 mL min^-1^. Ferricytochrome *c*, 0.5 mM, was dissolved in water and adjusted to pH 2 with HCl. This solution was rapidly mixed with a buffer of 50 mM phosphate and 50 mM acetate, pH 6.08, yielding a final pH after mixing of 4.54 and a final concentration of 0.25 mM cytochrome *c*. The time-zero spectrum used as a reference to calculate the reaction progress was prepared by mixing two solutions of 0.25 mM cytochrome *c* at pH 2.

### Hardware

Various parts have been machined in the laboratory’s workshop. Precision outlining and construction were done with the help of a long-working distance microscope (10 cm, 15x magnification). All components are mounted on a 1.5 cm thick stainless-steel base plate.

The cuvette-holder is made from UV transparent PMMA (polymethylmethacrylate) and black plexiglass. Light enters and exits the assembly through the PMMA. Three-axes manipulators, a plano-convex and bi-convex lens and lens holder are from Newport Corporation. The monochromator is equipped with a 75 Watt Xenon short-arc light source (Ushio, purchased from Optical Building Blocks Corporation, OBB). It runs under the Moco software program provided by OBB. The Newton DU-940N-BU CCD camera was purchased from Andor. Data are acquired with the Andor Solis imaging software, which runs the CCD detector. Fluid flow is initiated manually whereafter the CCD camera is activated. The CCD software is used to trigger the Moco program to initiate the monochromator. The CCD camera, cuvette holder and lenses are contained in a lightproof box. The CCD camera settings are as follows: Acquisition mode ‘kinetic series’, data type ‘counts’, frequency of 50 Hz (50 scans sec^-1^), vertical shift speed of 7.425 μs, pixel readout rate of 2.5 MHz, temperature of the CCD -60°C, number of active pixel rows 200 (see below), number of accumulated scans 5. The cycle time of 20 ms yields an illumination time of 220 μs per cycle. In each scan of 20 ms five cycles are recorded and added, i.e. the total dark time is 18.9 ms, the total illumination time 1.1 ms.

### Data analysis

Before analysis of UV-vis spectra, the background counts were subtracted from the light counts of sample and reference. Spectra were analyzed using two-component analysis [5], by fitting to the equation f(*x*,*y*) = *a·x* + *b·y* + *c*, in which *x* and *y* correspond to the one-component UV-vis spectra, i.e. oxidized cytochrome *c* and reduced cytochrome *c* for the reaction between ferrocytochrome *c* and sodium ferrihexacyanide, or, in the case of HPTS, the acid, and base forms, respectively. The coefficients *a* and *b*, which indicate the degree of conversion, vary between 0 and 1 (the maximal amount or concentration of a component); coefficient *c* is a vertical offset (and determined in all analyses <0.01). The three coefficients are calculated using linear regression with the starting and final component UV-vis spectra (oxidized cytochrome *c* and reduced cytochrome *c*, or the acid and base forms of HPTS) as the input basis set. These basis UV-vis spectra were recorded separately from the spectra recorded in the kinetic experiment, although they could be determined to good approximation from the initial and the final spectrum of a kinetic experiment. The advantage of using the whole spectrum for analysis rather than a single wavelength (i.e. 550 nm for cytochrome *c*) is that the whole spectrum contains ~300 spectral data points, which greatly improves the reliability of the determination of the degree of conversion and produces kinetic traces with much better signal-to-noise. The kinetic traces thus obtained are fitted to equations describing irreversible first-order or second-order reactions. The second order equation is given by:

$$\Delta ABS(t)=offset+Abs_{inf}+\frac{(B_{0}-A_{0})\cdot \epsilon \cdot b}{(B_{0}-A_{0})\cdot e^{(B_{0}-A_{0})\cdot t\cdot k_{2nd}}-1}$$ (1)

in which *offset* is a free parameter, *Abs*_*inf*_ is absorbance at infinite time, *ϵ* the extinction coefficient of ferrocytochrome *c*, and *b* is the optical path length in cm. *A*_*0*_ and *B*_*0*_ are initial concentrations of ferrocytochrome *c* and sodiumferrihexacyanide in M, *t* is time in seconds, *k*_*2nd*_ the second order rate constant in M^1^s^-1^.

Absorption data were baseline corrected, arranged in a wavelength versus time matrix, and subjected to singular value decomposition analysis with programs written in LabVIEW following the theoretical guidelines in [6], to determine the individual spectra of all intermediates and the time constants τ (i.e. inverse of the first-order rate constants, k) connecting them. The uncertainty in these τ values was estimated as follows: the root mean square error (RMSE) values between the reconstructed and the modeled time traces for each of the four traces were added up to a single sum RMSE value. Then the three k-values in the model were modified by a certain percentage and the sum RMSE value was calculated between model and modified model. Equality between this sum RMSE (between model and modified model) and the previous sum RMSE (between model and experimental data) value determined a general percentage uncertainty in the k values and therefore approximately so in the τ values. The found uncertainty was circa ±20%.

## Results

### The mixing device

**Fig 1:**
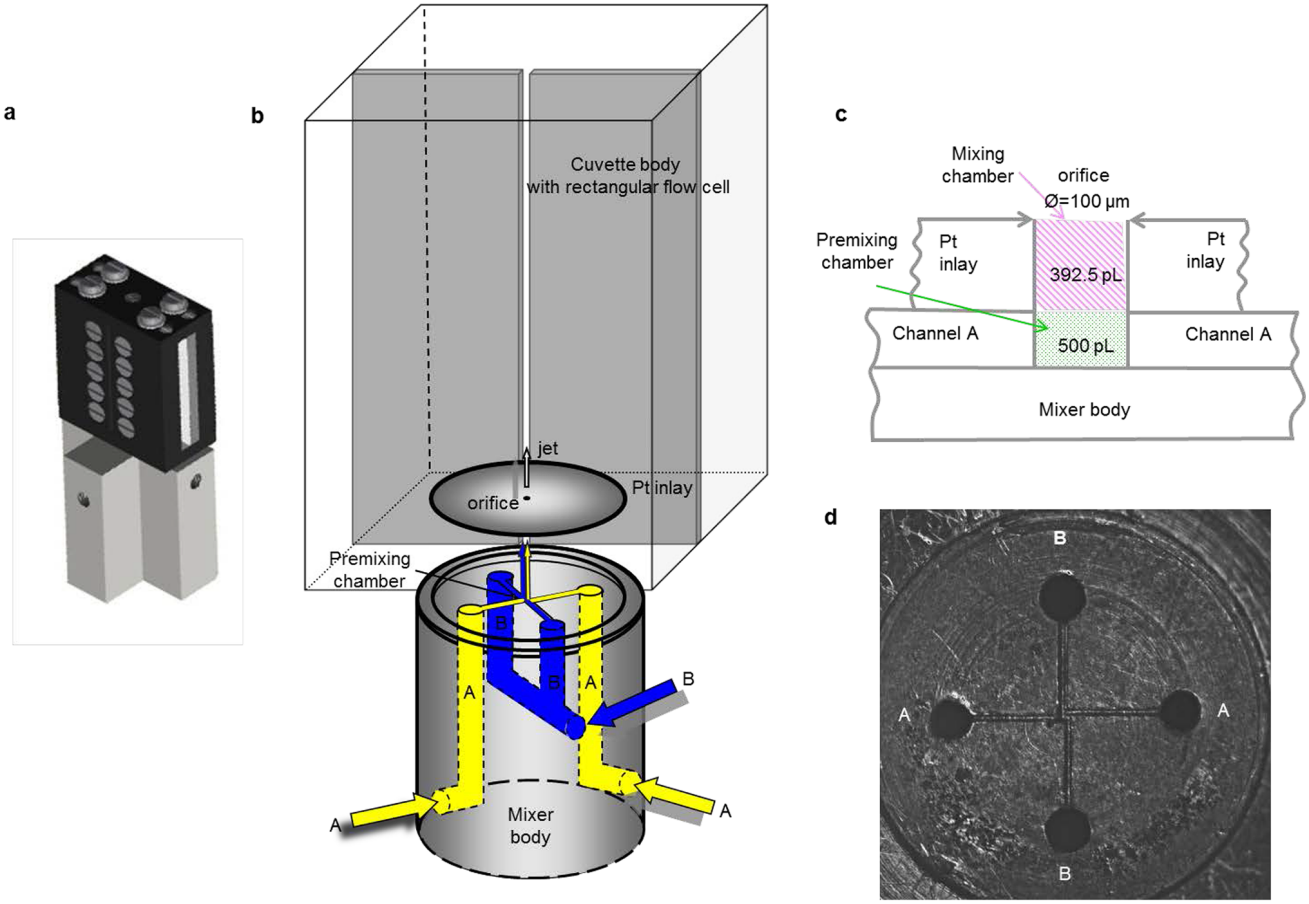
Design and geometry of the four-jet tangential ABAB micro-mixer. (a) Design of the micro-mixer body and the cuvette holder. (b) Geometry of the micro-mixer and the rectangular cuvette integrated in the plexiglass holder. Reaction components, A and B, enter the mixer body as indicated and subsequently flow through the four channels arranged in a cross. The components are then forced through a 100 μm wide and 50 μm deep μm Pt inlay mixing chamber (392.5 pL in volume and 3 mm in outer diameter). (c) Side-view slice through the mixer body and the Pt inlay along two opposite channels. (d) Top view of the channel arrangement and the premixing chamber of the dismounted mixer. The premixing chamber has dimensions of 100 x 100 x 50 μm^3^ yielding a volume of 500 pL.

**Fig 2:**
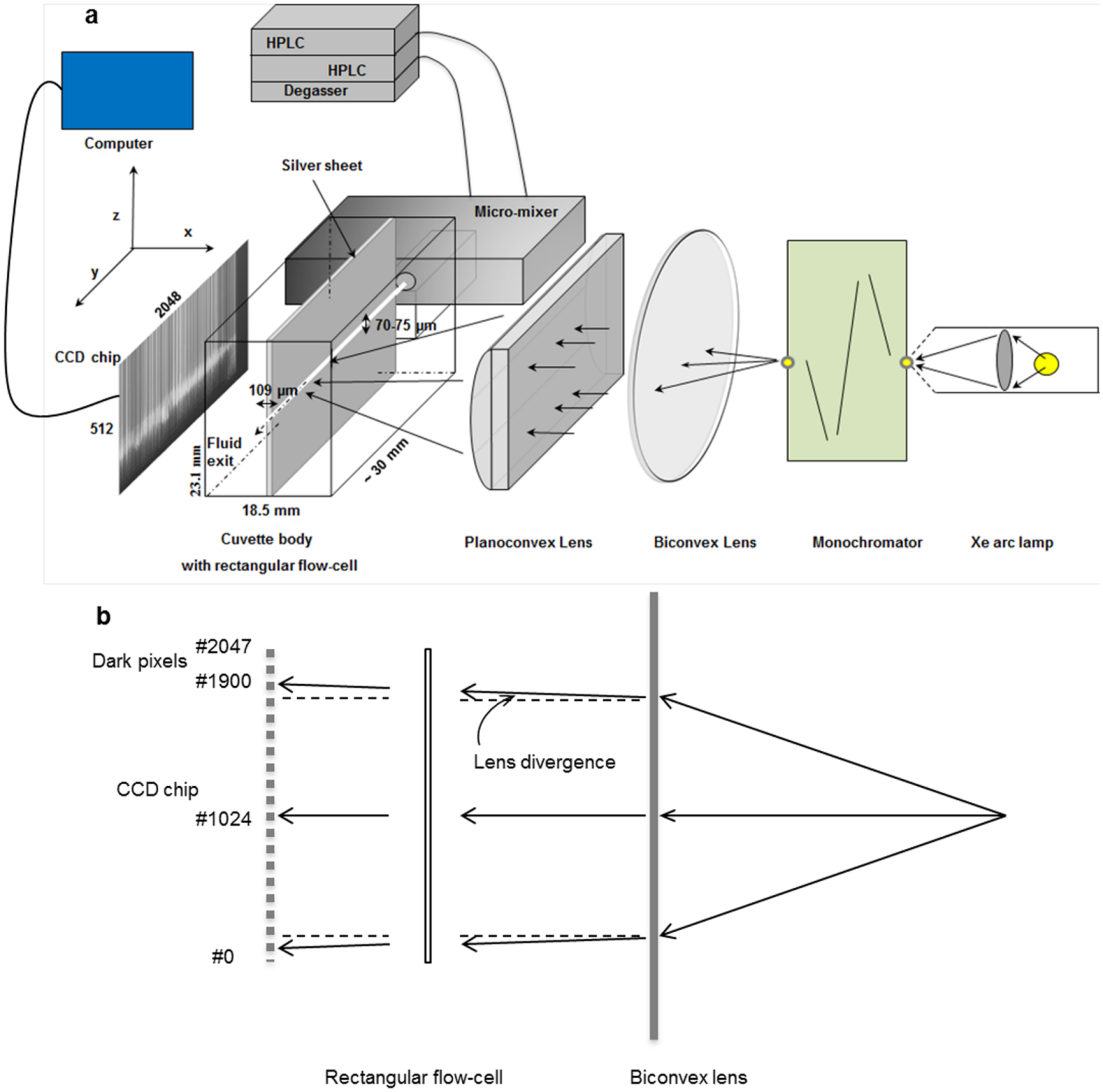
Schematic drawing of the optical components. (a) Monochromatic light is produced by a horizontally placed Xenon arc lamp, fitted to a scanning monochromator. The divergent monochromatic light is collimated by a bi-convex lens (150 mm focal length, 50 mm diameter) and focused along the length of the rectangular flow-cell by a plano-convex lens (100 mm focal length, 25 x 50 mm). The two parallel sheets of silver foil, each with a thickness of 109 μm (the optical path length), and 11.515 mm in height, are vertically separated by 70 ± 5 μm. The two sheets of silver are held in position by horizontal screws that press together the two plexiglass halves that make up the cuvette body. The rectangular CCD chip measures 512 × 2048 pixels (height × length; each pixel is 13.5 × 13.5 μm). The CCD camera and the scanning motor of the monochromator are synchronized by the computer. Reactants are delivered via the injection ports of two HPLC pumps to the stainless-steel micro-mixer and eventually to the flow-cell. The two three-axes manipulators to outline the various components, and the lightproof black box are omitted from the figure. The various components are not drawn to scale. The image of the rectangular cuvette recorded by the CCD camera in ‘image mode’ is shown in the figure. (b) Top view of the light paths through the lens and the rectangular cuvette indicating the effect of lens divergence on a collimated beam.

We describe the different components of the continuous-flow spectrometer with reference to Figs 1 and 2. In brief, the instrument consists of a (i) sample delivery system, (ii) a special mixer, which is tightly connected to (iii) a special cuvette housing, (iv) a xenon light source with a scanning monochromator and additional optics, (v) a CCD camera, and (vi) a triggering and processing computer. We have reported on an earlier version of the mixing device as part of of a rapid-freeze machine [7]. Also, we have earlier analyzed fundamental properties of the present mixer in the form of a transparent glass-silicon replica [8]. Additional description (S1–S3 Technical drawing) is available to support reconstruction of the instrument in other laboratories. Note that the authors have presently no intention to commercialize the spectrometer.

An outline of the mixer plus cuvette construct is given in Fig 1a. Two reactants, A and B, are delivered from sample bottles by two HPLC pumps (i.e. flow initiation is pneumatically) into the mixer body. Each reactant enters two opposite channels (∅ 300 μm in diameter) before meeting at a cross section, ‘the pre-mixing chamber’ (Fig 1b–1d). The four micro-channels leading to the pre-mixing chamber are 50 μm wide and deep. The opposite micro-channels are offset with respect to each other by a full width of the channel to induce a vortex of the four liquid streams by which mixing is enhanced (Fig 1d). In the pre-mixing chamber the reactants are pre-mixed at relatively low Reynolds numbers, Re (see below). After the pre-mixing chamber, the reactants are forced into the mixing chamber perpendicular to the plane of the channels, i.e. into the hole in the Pt inlay (100 μm orifice, 3 mm in outer diameter, 50 μm long and 0.39 nL in volume, made in house). Here the linear velocity of the liquids is highest and hence the Re leading to complete mixing. By increasing the flow rate, the residence time of the liquids in the pre-mixing- and the mixing chamber—the dead volume—becomes shorter. The minimal residence time in the dead volume, or dead time, is obtained at the highest flow rate of 20 mL min^-1^ (or 0.33 nL μs^-1^), and is calculated as 1.5 μs and 1.2 μs for the pre-mixing chamber and mixing chamber, respectively, totaling 2.7 μs. The Pt inlay is precisely positioned in front of the rectangular cuvette (Fig 1a and 1b) and acts both as a tight seal to prevent leaking of the reactants (up to 400 bar generated by the fast flow of the fluids) and as the micro compartment where mixing is completed. After the mixing chamber, the mixed reactants directly enter the optically transparent rectangular flow-cell integrated in the cuvette holder where UV-vis measurements are made (Figs 1b and 2a).

### Continuous-flow cuvette

The rectangular cuvette of 109 μm path length is integrated in the cuvette holder (Figs 1b and 2a). The rectangular flow channel is ~30 mm long and constructed from two parallel sheets of silver foil each of 109 μm thickness, separated edge-to-edge by 70 ± 5 μm. The silver foil acts as a seal to prevent leakage of the pressurized fluid (up to ~400 bar) and also acts as an effective shield to reduce stray light thus producing a spectrophotometer that is linear up to an absorbance of at least 3.5. After leaving the cuvette the fluid flows through a wide bore flexible tubing and is finally dispensed in a waste container.

### CCD detector

A fast CCD (charge-coupled device) detector rather than a photomultiplier/photodiode is used to record spectra. In a single run of the monochromator circa 2000 spectra are recorded, one in each vertically binned pixel array (z-axis, Fig 2a) corresponding to one spectrum per pixel on the y-axis. In the kinetic mode of the setup the different pixels along the y-axis of the CCD correspond to different reaction times (Fig 2a). The reaction time is determined by the distance between the mixer exit—recorded as ‘the first pixel’—and a given pixel, as well as by the linear flow rate of the liquid. At the highest flow rate of 20 mL min^-1^ the time resolution equals 350 ns per pixel resulting in a total time span of ~600 μs.

Two three-axes manipulators (an xyz linear stage and a two-axis tilt and rotation stage) were machined to fit on top of one another and adapted to carry the micro-mixer plus cuvette holder, enabling alignment of the flow-cell in the light path between the high-speed scanning monochromator and the CCD camera (Fig 2a). The CCD camera is next aligned to the flow-cell. The CCD camera is mounted on a small table allowing manual translations in the xy plane; vertical manual translations of the four supports are performed with four fine screws.

Since the cuvette and the CCD chip are approximately 22 mm apart, and the image of the 70 μm wide cuvette appears as 2.7 mm wide, the vertical divergence due to light scattering from the cuvette is approximately 0.12 (Fig 2b). To minimize vertical divergence and to block out stray light, the image of the flow-cell is positioned at the bottom 200 pixels; the camera is shielded by a black cover leaving only the bottom 200 pixels accessible to light. Data are then recorded in the full vertical binning, or crop 200, mode, which not only speeds up the recording of data, but also selects as much as possible only the fraction of absorbed light and not scattered light. The former is later processed by taking the average of these 200 pixels at each position in the y-direction of the camera. An important issue related to the time resolution is how well the y-axis (the time axis) is projected on the CCD chip as the cuvette and CCD are 22 mm apart. Collimation by a lens is never perfect (Fig 2b): with the monochromator exit slit width of 0.1 mm, the light intensity distribution spreads ±1 pixel in the y-axis direction of the CCD, which corresponds to an uncertainty of ±0.3 μs in time at the highest flow rate of 20 mL min^-1^. The divergence in the direction of the z-axis is irrelevant since the light intensity data are recorded in crop mode, and each column of the CCD camera is represented by a single light intensity value.

### Alignment of the setup and recording of spectra

In order to precisely align the setup, light of 474 nm is used, the maximum of the emission spectrum of the Xenon lamp, and the flow-cell is filled with milli-Q water. The various components are outlined until the image of the flow-cell is projected approximately in the middle of the CCD camera operating in image mode. The cuvette holder is manipulated precisely parallel to the bottom of the base plate using a mini leveler and perpendicular to the light beam, i.e. to a position where the ‘first pixel’ has a minimum peak width. The height and tilt of the CCD camera are subsequently adjusted with respect to the image of the flow-cell so that the latter is precisely horizontally aligned with the CCD chip. Finally, the CCD camera is elevated so that the image of the flow-cell is projected on the lowest 200 vertical pixels of the CCD chip and a cover, that acts as a permanent shutter, is placed over the front of the CCD camera so that only the lower 200 pixels are illuminated.

UV-vis spectra from 350–650 nm or part of this range are recorded with an exit slit of 0.1 mm (0.4 nm) and an entrance slit of 0.95 mm (3.8 nm) of the monochromator. Kinetic data are recorded at 50 nm sec^-1^ and the recording speed of the CCD camera is adjusted so that each recording cycle takes 20 ms. This produces UV-vis spectra with a data resolution of 1 nm; the spectral resolution determined by the exit slit is 0.4 nm.

### Optical quality

In order to record absorbance spectra with a single beam UV-vis spectrometer (Fig 2a) separate reference (*I*_*ref*_) and sample (*I*_*sam*_) transmission spectra are recorded and the absorbance spectrum is calculated as: *A* = -log (*I*_sample_/*I*_reference_). We tested the proportionality between absorbance and concentration of reduced cytochrome *c*, as expressed by the law of Lambert-Beer (S1 Fig). The UV-vis spectrometer is linear up to an absorbance of at least 3.5. Between 300–580 nm the spectra were found within experimental error to be the same to those determined in commercial spectrophotometers, including the ratio of the Soret-band to α-band absorbance of 5–5.3 (S1b Fig). The noise in each single spectrum, i.e. recorded in a vertically binned single-pixel position, equals ±0.0003 under the specific conditions of recording (single scan at 50 nm s^-1^, five cycles per data point, cf Materials and methods).

Absorbance spectra of ferro- and ferricytochrome *c* without fluid flow and with a fluid flow rate of 20 mL min^-1^ were recorded and found to be essentially identical (S2 Fig). For ferrocytochrome *c* the intensity of the α-band slightly decreases while that for the Soret-band slightly increases. The reason for these small spectral changes remains unclear, but it is not due to the pressure in the system (maximum ~260 bar) since spectra of cytochrome *c* are unaffected by pressures up to 6 kbar or 60 MPa [9].

### Efficiency of the micro-mixer

To determine the mixing efficiency of mixing devices, a chemical reaction must be completed within the mixing dead-time of the setup. Most suitable for this purpose are the fast protonation/deprotonation reactions with pH-sensitive dyes. HPTS (8-hydroxypyrene-1,3,6-trisulfonic acid, also known as pyranine) is a highly water-soluble pH-sensitive dye with a pK_a_ of ~7.3 in aqueous solution. The protonation and deprotonation rate constants are 1.8 10^11^ M^-1^ s^-1^ and 1–2 10^10^ M^-1^ s^-1^, respectively [10,11]. So under our experimental conditions ([H^+^] or [OH^-^] ~10 mM) protonation/deprotonation of HPTS occurs well within the time resolution of 350 ns per pixel at 20 mL min^-1^ fluid flow rate, and the mixing efficiency can thus be determined from the ratio of protonated versus non-protonated HPTS in the first few points of observation immediately after the liquids have left the Pt inlay and enter the cuvette.

**Fig 3:**
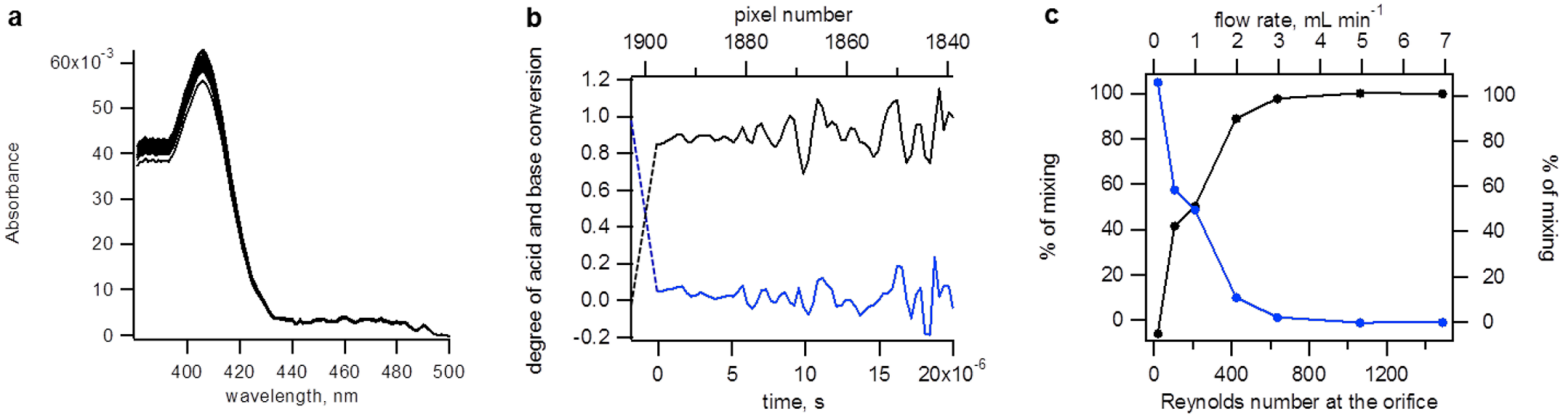
Determination of the mixer efficiency with a fast base-to-acid reaction. 3 mM HPTS in 20 mM NaOH (pH 12.3) was mixed with 30 mM HCl (pH 1.52). (a) Absorbance spectra of the reaction recorded within the first 7 μs (20 pixels) after the dead time at a liquid flow rate of 20 mL min^-1^. (b) Kinetic traces of the reaction determined in the first 64 pixels (22.4 μs after the dead time) at a liquid flow rate of 20 mL min^-1^. The spectra were decomposed using two-component analysis. Their normalized intensities are found to be close to one and zero, respectively, from the first data point onwards, indicating complete conversion from the base to acid form of HPTS. (c) Percentage of mixing in the first time point of the cuvette as a function of the flow rate and the Reynolds number calculated for the 100 μm Pt inlay orifice. Percentage of mixing is presented as appearance of acid (black) and disappearance of base (blue).

At the highest flow rate of 20 mL min^-1^ mixing is complete in the very first time point after mixing in the (pre-)mixing chambers evidenced by the appearance of only one spectral component in the first read-out pixel which is number 1899 (Fig 3a and S3b Fig). Either the ‘acidic’ 406 nm peak is found for the base to acid reaction (Fig 3a), or the ‘basic’ 458 nm peak for the acid to base reaction (S3b Fig). The residence time in the pre-mixing and mixing chambers (i.e. the dead volume) at a flow rate of 20 mL min^-1^ is 2.7 μs, hence mixing is completed well within the instrumental dead-time of 3.8 ± 0.3 μs.

To determine at which Reynolds number the mixing is completed at the very first point of observation, the base to acid and acid to base experiments were repeated at flow rates between 0.5–7 mL min^-1^ using a 100 μm Pt inlay. The mixing is completed already at a flow rate of approximately 5 mL min^-1^ at which the calculated *Re* in the 100 μm Pt inlay orifice is 1060 (Fig 3c and S3d Fig). This value is considerably lower than the onset of turbulence in pipe flow, which occurs at *Re* = 2040 [12]. That mixing is apparently complete in a pre-turbulent regime is possibly due to the vortex experienced by the four tangential liquid flows, which is induced by the channel offset.

### Determination of the dead-time of the mixer

To determine the dead-time of the setup, the fast, outer-sphere electron-transfer between ferrocytochrome *c* and sodium ferrihexacyanide was studied as a model reaction:

$$Cytochrome\;c^{2+}+{\left[Fe^{III}{\left(CN\right)}_{6}\right]}^{3-}⇄Cytochrome\;c^{3+}+{\left[Fe^{II}{\left(CN\right)}_{6}\right]}^{4-}$$ (2)

The second order rate constant of this reaction has been determined using stopped-flow kinetic measurements [13]. The rate constants at pH 7 reported in the literature vary between 0.87 x 10^7^ M^-1^ s^-1^ and 1.2 x 10^7^ M^-1^ s^-1^. At concentrations of 100 mM sodium ferrihexacyanide the turnover frequency is approximately 1 μs, suitable for our purposes. Given the spread in the reported rate constants, we re-determined the rate constant for the reaction by the stopped-flow method at temperatures of 19.3°C and 34.4°C (S4 Fig, and S1 Table).

**Fig 4:**
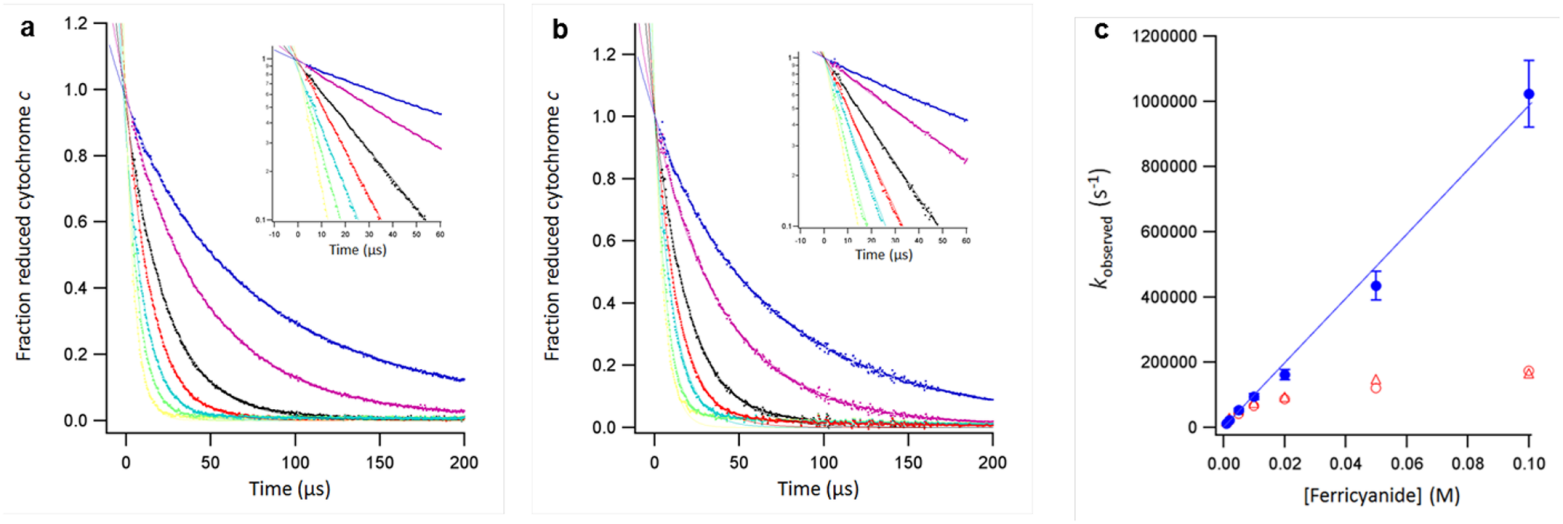
Determination of the overall dead time with a fast electron-transfer reaction. Ferrocytochrome *c* (0.48 mM) was reacted with various concentrations of sodium ferrihexacyanide at 27.5°C. Kinetic traces of the reaction show the data after two-component analysis as the fraction of ferrocytochrome *c* vs time and after calculating the logarithm of this fraction (inset) for the Soret region 352–450 nm (a); and the α-band region 500–600 nm (b). The buffer was 50 mM potassium phosphate (pH 7, ionic strength is 0.1 M). Final ferrihexacyanide concentrations were: 1, 2, 5, 10, 20, 50 and 100 mM (from blue to yellow). The lines through the data points are second-order fits (1 and 2 mM ferrihexacyanide) and first-order exponentials. The fits all cross in a single point (±1 pixel or ±0.3 μs) where the fraction of reduced cytochrome *c* equals 1. Experimental data are shown with a time offset of 3.8 μs, i.e. the determined dead time. (c) Observed rates (*k*_observed_) as a function of the ferrihexacyanide concentration. Red Δ are from analysis of the Soret band, red o are from the α-band. Blue ● are the observed rate constants after corrections for the ionic strength (see text) and are the average of the analyses performed for the Soret band and α-band; The blue line is a linear fit through the origin and the data points yielding a second-order rate constant of 0.986 ± 0.028 10^7^ M^-1^s^-1^.

To calculate the rate constant and the instrumental dead-time, the reaction was studied with excess sodium ferrihexacyanide over ferrocytochrome c under both first- and second-order conditions (Fig 4). Analysis of the traces indicated that *k*_*obs*_ was not proportional to the concentration of ferrihexacyanide above approximately 10 mM (Fig 4c, red data points). Therefore, the calculated second-order rate constant is not constant and was found to decrease by circa a factor of ten, i.e. from ~10^7^ to 10^6^ M^-1^s^-1^ when the concentration of ferrihexacyanide was increased from 1 mM to 100 mM. However, the increase in the concentration of oxidant leads to an increase in the ionic strength and this will reduce the rate of the reaction because it involves two oppositely charged reactants (Eq 3). The oxidant ferrihexacyanide carries a high net negative charge of -3 (*Z*_*1*_ = -3) and hence the total ionic strength increases from 0.1 M to 0.7 M in the ferrihexacyanide concentration range from 1 mM to 100 mM; the phosphate buffer contributes 0.094 M to the ionic strength. The total charge of reduced cytochrome c is +6.5 (*Z*_*2*_ = +6.5) [14]. The effect of ionic strength on the rate of electron transfer reactions is well described in the literature (Eq 16 in [14]):

$$lnk=lnk_{0}-3.567\cdot \left(\frac{e^{-kR_{1}}}{1+kR_{2}}+\frac{e^{-kR_{2}}}{1+kR_{1}}\right)\cdot \frac{Z_{1}Z_{2}}{R_{1+}R_{2}}$$ (3)

Herein the ionic radii *R*_*1*_ and *R*_*2*_ were taken as 4.5 Å and 16.6 Å for ferrihexacyanide and ferrocytochrome c [14]. The ionic strength parameter for water, *k*_*0*_ is equal to 0.329 μ^1/2^ Å^-1^ and *μ* is the ionic strength. The rate constant at zero ionic strength is k_0_. The second-order rate constants corrected for ionic strength are plotted in Fig 4c (blue data points and fit) and yield for 0.1 M ionic strength the value of *k* = 0.986 ± 0.028 x 10^7^ M^-1^s^-1^ (S1 Table). This value is within the range of values reported in the literature.

In two-component analysis we employed the spectra of ferri- and ferrocytochrome c as basis spectra (S5b Fig). Separate analyses were made for the Soret spectral region 352–450 nm (Fig 4a) and for the α-band 500–600 nm (Fig 4b). The second-order fits cross at a single time point where the fraction of the reactant, ferrocytochrome c, equals 1.0 (Fig 4a and 4b inserted panels). This time point represents the true ‘time zero’. Approximately 3.8 μs (or 11 pixels on the CCD camera) beyond nominal time zero, the second-order fits agree well with calculated fractions of reduced cytochrome c. The calculated dead-time of the instrument is thus 3.8 ± 0.3 μs. This value is consistent with the total dead volume of the micro-mixer of 0.9 nL, which is equivalent to 2.7 μs at a fluid flow rate of 20 mL min^-1^.

### Proof of principle: Cytochrome *c* refolding

Acid denatured cytochrome *c* at pH 2 refolds spontaneously when the pH is rapidly changed to a final pH 4.5 [15]. With fluorescence spectroscopy to monitor changes in Trp59 or single wavelength UV-vis absorbance spectroscopy in the Soret region two kinetic phases (τ = 57–59 μs and 430–500 μs) were previously observed in the first few milliseconds of the refolding reaction [2–4]. Althoug the rapid phase (τ = 58 μs) is close to their instrumental dead-time (45–50 μs), Roder et al. concluded from extrapolation of the kinetic traces to time zero, that this phase was not preceded by any earlier UV-vis or fluorescence observable folding event.

**Fig 5:**
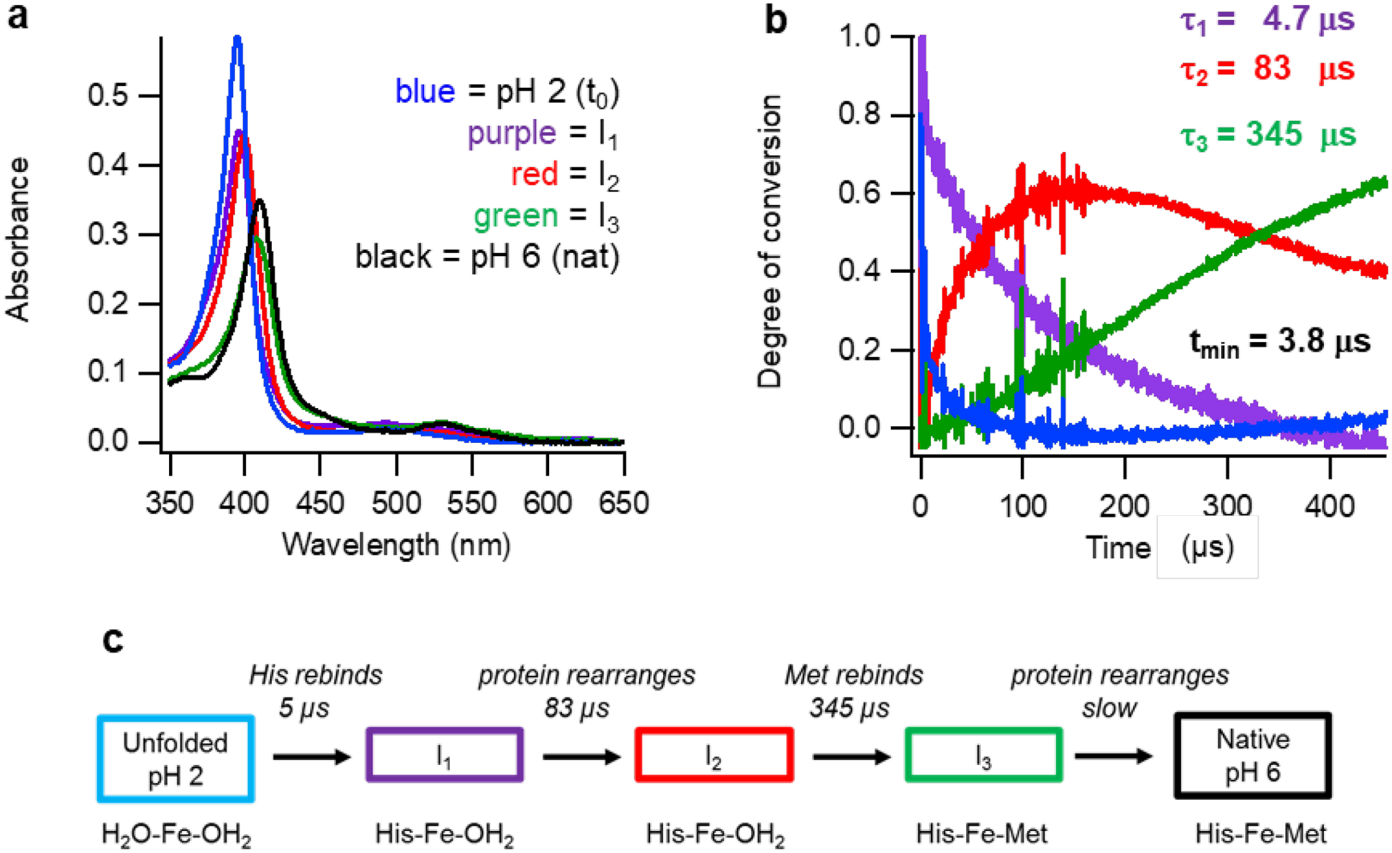
Analysis of early stages in the re-folding of acid-denatured cytochrome *c*. Spectral components (a) and kinetic traces (b) of cytochrome *c* in a pH-jump (2 to 4.5) experiment obtained from singular value decomposition analysis. Time zero (t_0_) at pH 2 (blue), I_1_ (magenta), I_2_ (red), I_3_ (green). The spectrum of native protein (nat) at pH 6 (black) was obtained after prolonged incubation. (c) Interpretational scheme of the experiment as described in the text.

We have used our new instrument to study the refolding kinetics of denatured cytochrome *c* by the same pH-jump technique as described by Roder. The experiment was performed at 20 mL min^-1^ fluid flow rate providing a total reaction window of 0.65 ms (Fig 5). Singular value decomposition analysis of the spectra obtained during the reaction yielded four significant spectral components. Three rate constants and four spectra were calculated according to the following model: U(Unfolded) → I_1_ → I_2_ → I_3_. Herein I_3_ is the final, partially folded, intermediate at pH 4.5; subsequent re-folding to the native state is a multiple-phase, slower process on a time scale of milliseconds to seconds [2,9,16,17]. After a very rapid initial phase (τ = 4.7 μs) not observed before, partial refolding proceeds with life times of 83 μs and 345 μs.

The Soret maxima of the three calculated spectral intermediates (I_1_, I_2_ and I_3_) progressively shift to the red during the reaction from 395 nm to 408 nm for I_3_, which is indicative of a full change from a high-spin configuration produced by two weak-field axial ligands, via a mixture of high-spin and low-spin due to a weak-field and an strong-field ligand, to a low-spin complex with two strong-field ligands [18].

## Discussion

The development of a mechanistic description of an enzyme in action, or a protein folding towards its native state, requires molecular-structural information as a function of time. An increase in time resolution adds significance to the derived structure-function relationship. Some particular processes, such as photosynthesis, may be triggered with a laser light switch, which in principle affords resolution of intermediates at the nanosecond or picosecond time scale. However, the more universally applicable switch-on by mixing reactants turbulently is constrained by mechanical and fluid-dynamical limitations, which typically (e.g., with commercial stopped-flow or rapid-freeze instruments) affords resolution on the millisecond time scale only. As the overall turnover rate of many enzymes is of this order or faster, the development of faster instrumentation is clearly of fundamental value. We have previously reported on the development of a rapid-mixing, rapid-freezing instrument with a one to two orders of magnitude increased time resolution and a dead time of circa 75 μs [1,7].

We have now turned to the development of a continuous-flow UV-visible spectrometer to push the attainable time resolution a step further. The best value reported thus far is a dead time of circa 45–50 μs for a single-wavelength instrument [2–4]. We have now attained an order of magnitude better dead time for a fully scanning spectrometer.

Our instrument has a dead time of 3.8 ± 0.3 μs and it produces 1900 UV-vis spectra, here tested from 350–650 nm, separated in time by only 350 ns for a period of 650 μs at the flow rate of 20 mL min^-1^. The flow rate can be reduced to 5–7 mL min^-1^ with complete mixing in the first data point thus extending the time base to circa 2 ms, i.e. overlapping with that of stopped-flow setups. The optical sensitivity of the instrument is illustrated by a signal-to-noise ratio >50 for spectra recorded in a volume of only 110 pL with 10–90 femtomol of horse heart cytochrome *c*. The rectangular flow-cell constructed from two parallel sheets of silver metal affords an absorbance that is linear with concentration up to at least 3.5. The extinction coefficient as determined with regular, 1 cm path length, rectangular cuvettes applies using a path length of 109 μm. By averaging each data point five times the noise level in the absorbance is as low as 0.0003.

As a proof of principle we re-visited the refolding kinetics of acid denatured cytochrome *c* using the pH-jump technique. Three time constants and four spectra were determined representing the starting pH-denatured protein and its conversion into the three main components, I_1_-I_3_, formed in the first 650 μs of the reaction. The reaction was previously reported to consist of only two irreversible steps [2–4]. A very rapid initial phase (τ = 4.7 μs) was not observed before. The life times of 83 and 345 μs that we determined for the second and third phase are similar to the values of 57–59 and 430–500 μs reported by Roder et al. [2–4].

We offer the following mechanistic interpretation of this experiment: At the initial pH 2, the heme Fe^3+^ is high spin and it exhibits a Soret maximum at 395 nm and another band at 495 nm, suggesting coordination with two weak-field water ligands [19]. At pH 2 His18 is protonated and dissociated from the heme iron. Shifts in the Soret maximum between 397 nm and 400 nm are consistent with a change from high spin to a mixture of low spin and high spin due to coordination of one strong-field His18 ligand and one weak-field water ligand. Apparently, both intermediates I_1_ and I_2_ are mixtures of high-spin and low-spin configurations. From I_1_ to I_2_ the spectra hardly change, therefore we interpret this as a conformational adjustment after the His binding. From I_2_ to I_3_ the shift in the Soret maximum between 400 nm and 408 nm is consistent with a change to fully low spin, which must be due to the binding of another strong-field ligand: Met80. From I_3_ to the fully re-folded state at pH 6 there is again a small change presumably due to a conformational adjustment to the Met80 binding. The complete process is graphically summarized in the scheme in Fig 5c.

As a practical caveat we note that the continuous-flow approach requires large amounts of reagents, usually at millimolar concentrations, to obtain sufficient optical absorbance at a path length of 100 μm. The requirement of high amounts of reagents remains a general disadvantage of continuous-flow over stopped-flow methods. On the positive side we point out that the specifications of our spectrometer now for the first time allow for data acquisition in a time regime overlapping with that in which molecular dynamics calculations of reaction trajectories are routinely made to date (cf [20] and refs quoted therein).

## Supporting information

S1 Technical drawing 2D CAD drawing of the cuvette assembly. (PDF)

S2 Technical drawing 3D CAD rotatable drawing of the cuvette assembly. (PDF)

S3 Technical drawing A series of pictures of the spectrometer with indications of dimensions. (PDF)

S1 Fig Optical quality of ferrocytochrome c absorbance spectra at various concentrations. (a) Ferrocytochrome *c* concentrations were 0.001 (red), 0.002, 0.005, 0.01, 0.025, 0.05, 0.1, 0.2, 0.5, 1.0 and 2.0 (blue) mM, respectively. The spectra are the average of the spectra recorded in pixels 0–1900, along the whole length of the rectangular flow-cell, and were put arbitrarily at zero at 650 nm. (b) The relation between absorbance and concentration of ferrocytochrome *c* at 415 nm and 550 nm. The straight lines are fits to the data points. The ratio of the slopes of these lines equals 5.0, i.e. similar to the ratio of the extinction coefficients for the Soret- and α-band maxima determined in commercial UV-vis spectrometers.
(PDF)

S2 Fig Comparison between static absorbance spectra and spectra recorded during fluid flow. Absorbance spectra of ferro- and ferricytochrome *c* without fluid flow (blue) and with a fluid flow (red) rate of 20 mL min^-1^. Bottom spectra are ferricytochrome *c*. The spectra of ferrocytochrome *c* are offset by +0.5 for clarity. The spectra are the average of the spectra recorded in pixels 0–1900, along the whole length of the flow-cell.
(PDF)

S3 Fig HPTS acid to base reaction. (a) The reference absorbance spectra: acid control spectra (black: 3 mM HPTS in 10 mM HCl (pH 2) mixed vs 10 mM HCl) and base control spectra (blue: 3mM HPTS in 10 mM NaOH (pH 12) mixed vs 10 mM NaOH) recorded within the first 7 μs (20 pixels) after dead time at a liquid flow rate of 20 mL min^-1^. (b) Absorbance spectra of the reaction of 3 mM HPTS in 20 mM HCl (pH 1.7) mixed with 30 mM NaOH (pH 12.48) recorded within the first 7 μs (20 pixels) after dead time at a liquid flow rate of 20 mL min^-1^. (c) Kinetic traces of the reactions determined in the first 64 pixels (22.4 μs) after dead time at a liquid flow rate of 20 mL min^-1^. The spectra were analysed using two-component analysis. Their normalized intensities are found to be close to zero and one, respectively, from the first data point onwards, indicating complete conversion from the acid to base form of HPTS. (d) Percentage of mixing in the first time point of the rectangular cuvette as a function of the flow rate and the Reynolds number calculated for the 100 μm Pt inlay orifice. Percentage of mixing is presented as appearance of base (blue) and disappearance of acid (black).
(PDF)

S4 Fig Stopped-flow kinetic traces of the oxidation of ferrocytochrome *c* by various concentrations of sodium ferrihexacyanide. Ferrocytochrome *c* was 29.6 μM. Sodium ferrihexacyanide concentrations were 0.001 (red), 0.002, 0.003, 0.006, 0.01 and 0.02 (black) mM. The buffer was 50 mM potassium phosphate, pH 7.0. The black solid lines are second-order fits used to determine the rate constant of the reaction. The fraction of reduced cytochrome *c* has been plotted (data points) as calculated with two-component analysis from the absorption at 550 nm at a temperature of (a) 19.3°C and (b) 34.4°C.
(PDF)

S5 Fig Absorbance spectra of oxidation of cytochrome *c* by sodium ferrihexacyanide. 0.5 mM ferrocytochrome c and 20 mM sodium ferrihexcyanide were in 50 mM potassium phosphate buffer, pH 7.0. (a) Absorbance spectra recorded in the time span from 3.8 μs to 70 μs after initiation of the reaction. The spectrum with the largest amplitude (blue) at 415 and 550 nm represents the t = 0 sample, recorded separately. The figure shows 190 spectra, i.e. the time resolution of the spectra is 350 ns. (b) Selection of spectra of ferrocytochrome *c* in reaction with ferrihexacyanide (red) and comparison to the spectra of ferri- (black) and ferrocytochrome *c* (blue) that served as reference spectra to calculate the amount of conversion by component analysis.
(PDF)

S1 Table Rate constants at 0.1 M ionic strength for the reaction between ferrocytochrome *c* and sodium ferrihexacyanide. (PDF)

## References

[1] Mitic S., de Vries S. Rapid mixing techniques for the study of enzyme catalysis In: Egelman Edward H., editor: Comprehensive Biophysics Volume 1: Biophysical techniques for structural characterization of macromolecules. Academic Press; 2012 pp. 514–532.

[2] Shastry M.C., Luck S.D., Roder H. A. (1998) continuous-flow capillary mixing method to monitor reactions on the microsecond time scale. Biophys J. 1998; 74: 2714–2721. doi: 10.1016/S0006-3495(98)77977-9 9591695 10.1016/S0006-3495(98)77977-9 PMC1299611

[3] Roder H., Maki K., Cheng H. Early events in protein folding explored by rapid mixing methods. Chem Rev. 2006; 106: 1836–1861. doi: 10.1021/cr040430y 16683757 10.1021/cr040430y PMC2556641

[4] Shastry M.C., Roder H. Evidence for barrier-limited protein folding kinetics on the microsecond time scale. Nature Struct Biol. 1998; 5: 385–392. 9587001 10.1038/nsb0598-385

[5] Malinowski E.R. Factor Analysis in Chemistry. 3rd ed, Wiley; 2002.

[6] Henry E.R., Hofrichter J. Singular value decomposition: application to analysis of experimental data. Methods Enzymol. 1992; 210: 129–192.

[7] Cherepanov A.V., de Vries S. Microsecond freeze-hyperquenching: development of a new ultrafast micro-mixing and sampling technology and application to enzyme catalysis. Biochim Biophys Acta. 2004; 1656: 1–31. doi: 10.1016/j.bbabio.2004.02.006 15136155 10.1016/j.bbabio.2004.02.006

[8] Mitic S., van Nieuwkasteele JW, van den Berg A, de Vries S Design of turbulent tangential micro-mixers that mix liquids on the nanosecond time scale. Anal Biochem. 2015; 469: 19–26. doi: 10.1016/j.ab.2014.10.003 25447461 10.1016/j.ab.2014.10.003

[9] Takahashi S., Yeh S.-R., Das T.K., Chan C.-K., Gottfried D.S., Rousseau D.L. Folding of cytochrome *c* initiated by submillisecond mixing. Nature Struct Biol. 1997; 4: 44–50. 8989323 10.1038/nsb0197-44

[10] Spry D.B., Goun A., Fayer M.D. Deprotonation dynamics and Stokes shift of pyranine (HPTS). J Phys Chem A. 2007; 111: 230–237. doi: 10.1021/jp066041k 17214458 10.1021/jp066041k

[11] Mojumdar S.S., Mondal T., Das A.K., Dey S., Bhattacharyyaa K. Ultrafast and ultraslow proton transfer of pyranine in an ionic liquid microemulsion. J Chem Phys. 2010; 132: 194505 doi: 10.1063/1.3428669 20499977 10.1063/1.3428669

[12] Avila K., Moxey D., de Lozar A., Avila M., Barkley D., Hof B. The Onset of turbulence in pipe flow. Science. 2011; 333: 192–196. doi: 10.1126/science.1203223 21737736 10.1126/science.1203223

[13] Brand K.G., Parks P.C., Czerlinski G.H., Hess G.P. On the elucidation of the pH dependence of the oxidation-reduction potential of cytochrome *c* at alkaline pH. J Biol Chem. 1996; 241: 4180–4185. 4958912

[14] Rosenberg R.S., Wherland S., Holwerda R.A., Gray H.B. Ionic strength and pH effects on the rates of reduction of blue copper proteins by Fe(EDTA)^2-^. comparison of the reactivities of *Pseudomonas aeruginosa* azurin and bean plastocyanin with various redox agents. J Am Chem Soc. 1976; 98: 6364–6369. 9439 10.1021/ja00436a049

[15] Yeh S.-R., Han S., Rousseau D.L. Cytochrome *c* folding and unfolding: a biphasic mechanism. Acc Chem Res. 1998: 31: 727–736.

[16] Yeh S.-R., Takahashi S., Fan B., Rousseau D.L. Ligand exchange during cytochrome *c* folding. Nature Struct Biol. 1997; 4: 51–56. 8989324 10.1038/nsb0197-51

[17] Yeh S.-R., Rousseau D.L. Folding intermediates in cytochrome *c*. Nature Struct Biol. 1998; 5: 222–228. 9501916 10.1038/nsb0398-222

[18] Margoliash E., Schejter A. Cytochrome *c*. Adv Protein Chem. 1966; 21: 113–286. 5333288 10.1016/s0065-3233(08)60128-x

[19] Babul J., Stellwagen E. Participation of the protein ligands in the folding of cytochrome *c*. Biochemistry. 1972: 11: 1195–1200. 5062485 10.1021/bi00757a013

[20] Post C.B., Levy R.M. Editorial overview: theory & computation. Curr Opin Struct Biol. 2017; 43: iv–vi. doi: 10.1016/j.sbi.2017.04.008 28477855 10.1016/j.sbi.2017.04.008 PMC6225781

