# Supplementary material for: Microsecond time-scale kinetics of transient biochemical reactions
Source: PLoS One. 2017 Oct 3;12(10):e0185888. doi: 10.1371/journal.pone.0185888 (PMC5626514; doi:10.1371/journal.pone.0185888)
Supplement: S1 Technical drawing — (PDF) [file pone.0185888.s001.pdf]

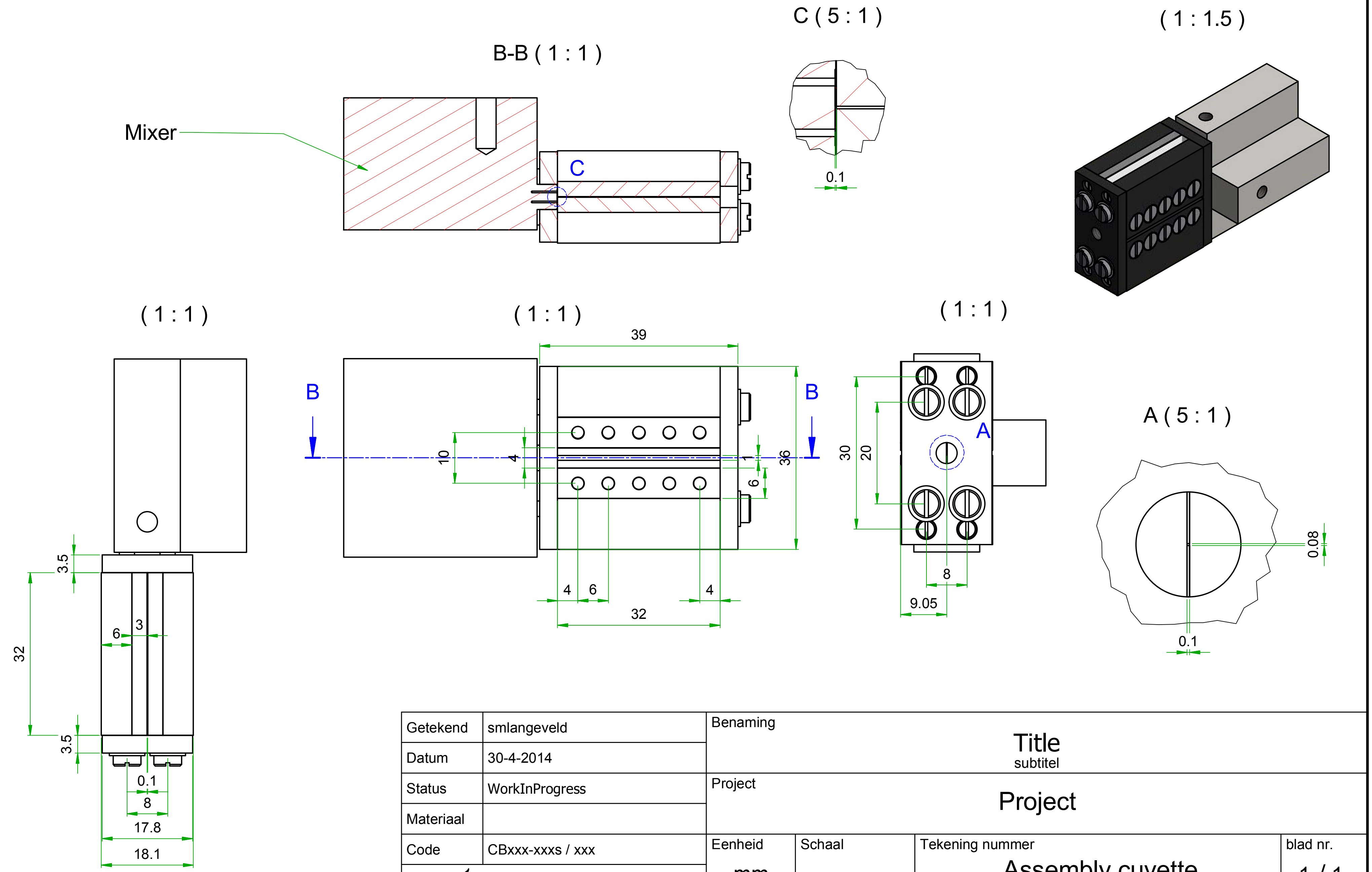

Getekend	smlangeveld	Benaming<br>Title<br>subtitel			
Datum	30-4-2014				
Status	WorkInProgress	Project<br>Project			
Materiaal					
Code	CBxxx-xxxs / xxx	Eenheid<br>mm	Schaal	Tekening nummer<br>Assembly cuvette	blad nr.<br>1 / 1
TU Delft Delft University of Technology<br>DEMO - CMO			Alle vlakken tenzij anders vermeld	Algemene toleranties en ruwheid tenzij anders vermeld volgens: NEN-ISO 1101, NEN-ISO 2768-m H-E en NPR 3634/3638<br>Auteursrecht voorbehouden volgens de Wet	Formaat<br>A4

M:\ud\demo\demo-shared\BIO\BT-CADshare\MARCEL\ENZ02-cuvette\onderdelen\Assembly cuvette.iam
