# Supplementary material for: Microsecond time-scale kinetics of transient biochemical reactions
Source: PLoS One. 2017 Oct 3;12(10):e0185888. doi: 10.1371/journal.pone.0185888 (PMC5626514; doi:10.1371/journal.pone.0185888)
Supplement: S2 Technical drawing — (PDF) [file pone.0185888.s002.pdf]

Assembly cuvette

TU Delft DEMO
www.demo.tudelft.nl

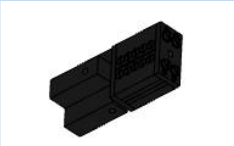

Master

PROJECT	Project
DESIGNER	smlangeveld
