# Supplementary material for: Microsecond time-scale kinetics of transient biochemical reactions
Source: PLoS One. 2017 Oct 3;12(10):e0185888. doi: 10.1371/journal.pone.0185888 (PMC5626514; doi:10.1371/journal.pone.0185888)
Supplement: S3 Technical drawing — (PDF) [file pone.0185888.s003.pdf]

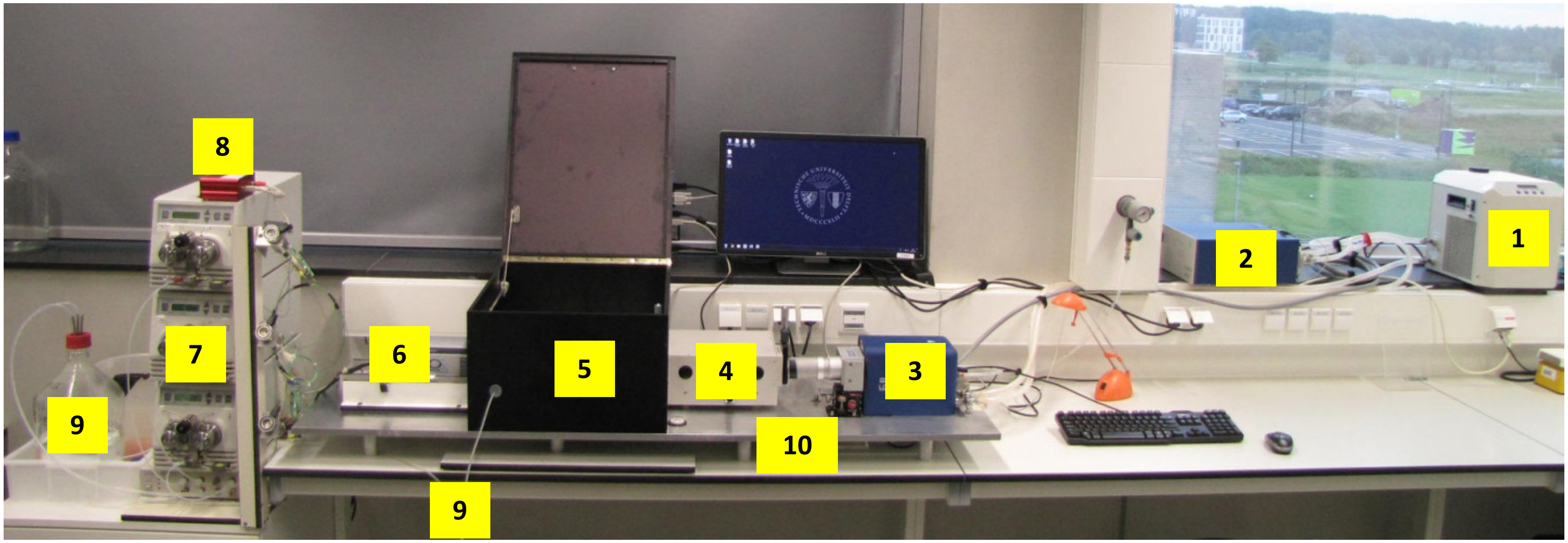

Picture 1: overview of setup

(1) Thermostat of lamp
(2) Power supply of lamp
(3) Xenon lamp
(4) Scanning monochromator
(5) Enclosure of central optics (l × w × h = 55 × 42 × 26 cm)
(6) CCD camera
(7) HPLC pumps (with injection ports on the right)
(8) Trigger
(9) Waste
(10) Base plate (l × w = 55 × 153 cm)

Picture 2: front view

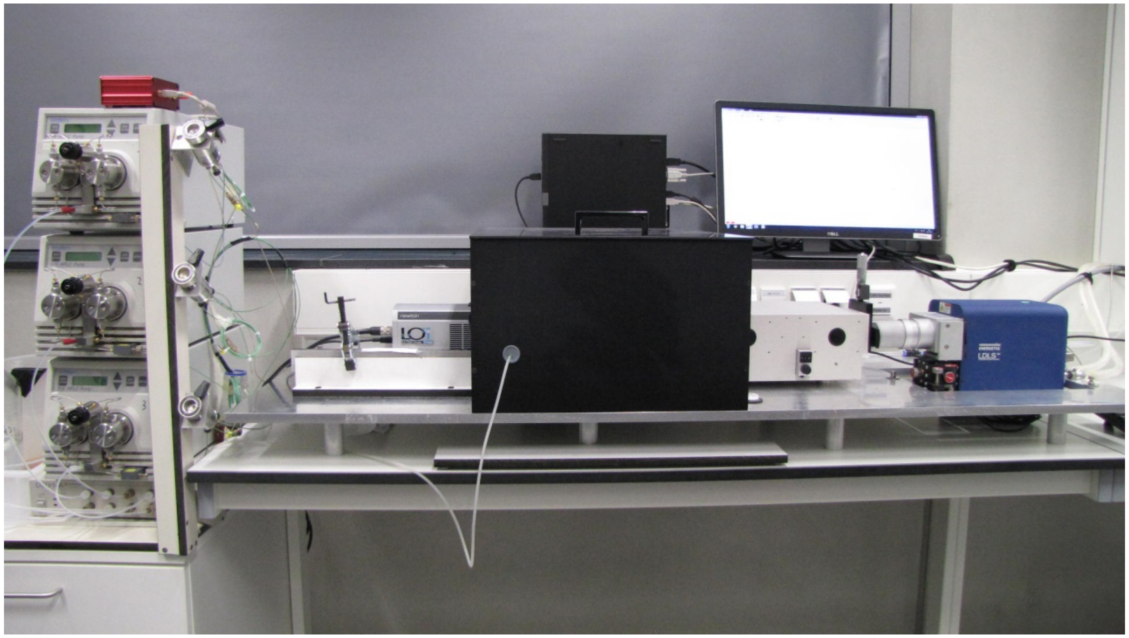

Picture 3: top view

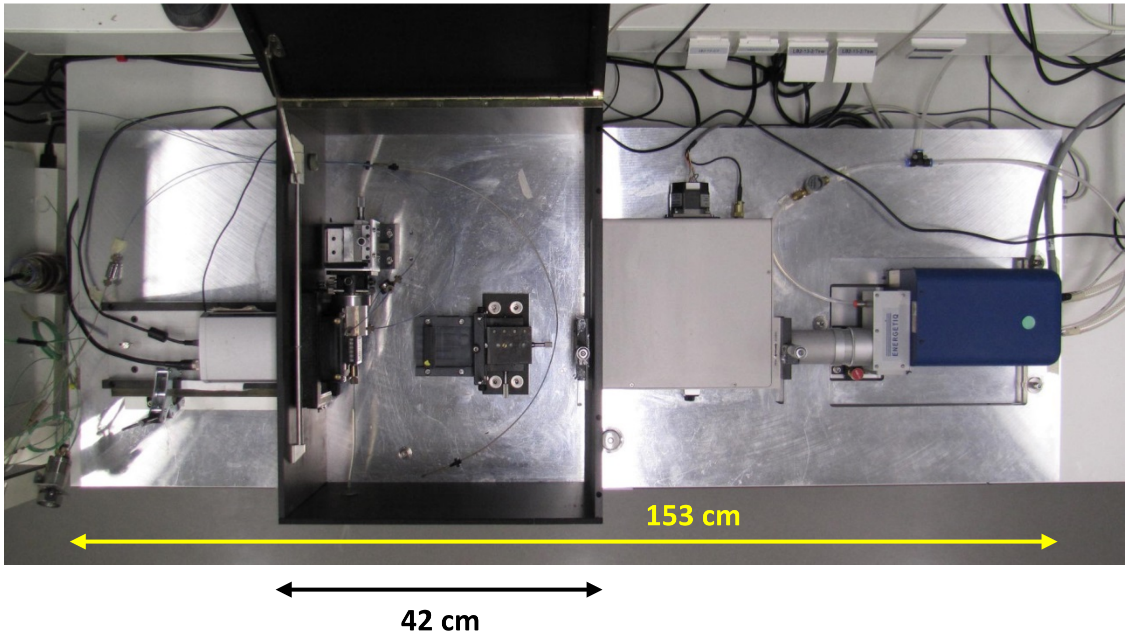

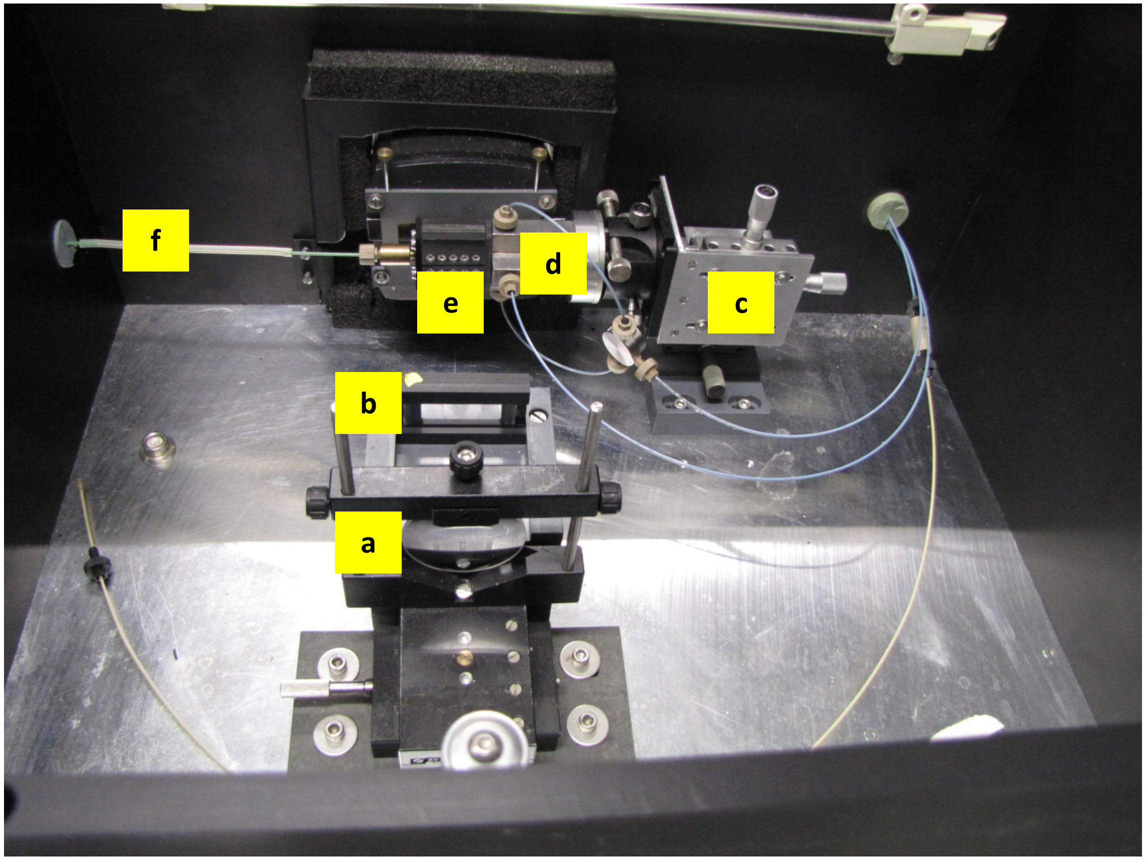

Picture 4: central optics (view direction from xenon lamp to CCD camera)

(a) Bi-convex lens
(b) Plano-convex lens
(c) 3D stage
(d) Mixer housing
(e) Cuvette housing
(f) Waste
