# Supplementary material for: Microsecond time-scale kinetics of transient biochemical reactions
Source: PLoS One. 2017 Oct 3;12(10):e0185888. doi: 10.1371/journal.pone.0185888 (PMC5626514; doi:10.1371/journal.pone.0185888)
Supplement: S1 Fig — (a) Ferrocytochrome c concentrations were 0.001 (red), 0.002, 0.005, 0.01, 0.025, 0.05, 0.1, 0.2, 0.5, 1.0 and 2.0 (blue) mM, respectively. The spectra are the average of the spectra recorded in pixels 0–1900, along the whole length of the rectangular flow-cell, and were put arbitrarily at zero at 650 nm. (b) The relation between absorbance and concentration of ferrocytochrome c at 415 nm and 550 nm. The straight lines are fits to the data points. The ratio of the slopes of these lines equals 5.0, i.e. similar to the ratio of the extinction coefficients for the Soret- and α-band maxima determined in commercial UV-vis spectrometers. (PDF) [file pone.0185888.s004.pdf]

S1 Fig. Optical quality of ferrocytochrome *c* absorbance spectra at various concentrations.

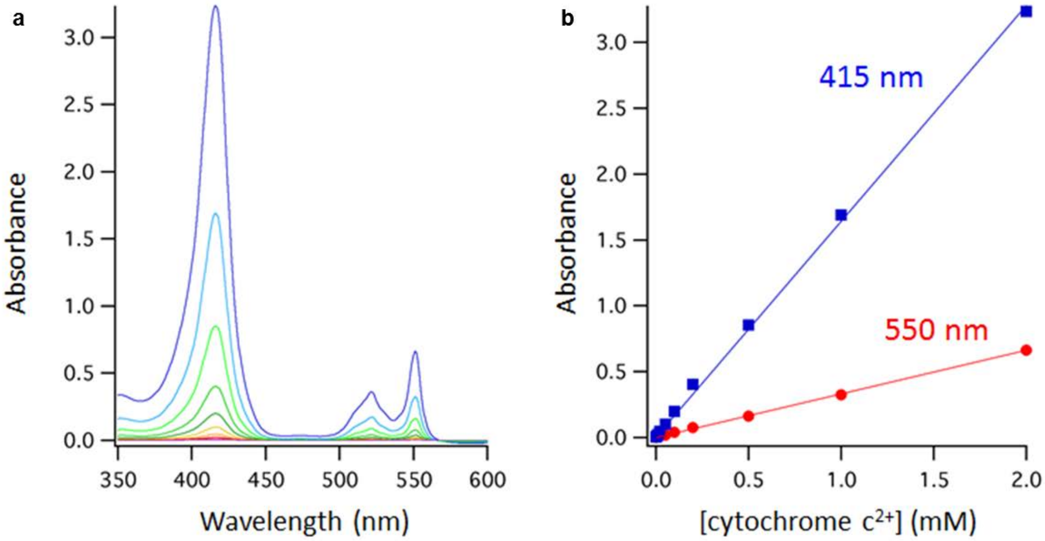

(a) Ferrocytochrome *c* concentrations were 0.001 (red), 0.002, 0.005, 0.01, 0.025, 0.05, 0.1, 0.2, 0.5, 1.0 and 2.0 (blue) mM, respectively. The spectra are the average of the spectra recorded in pixels 0 – 1900, along the whole length of the rectangular flow-cell, and were put arbitrarily at zero at 650 nm. (b) The relation between absorbance and concentration of ferrocytochrome *c* at 415 nm and 550 nm. The straight lines are fits to the data points. The ratio of the slopes of these lines equals 5.0, i.e. similar to the ratio of the extinction coefficients for the Soret- and α-band maxima determined in commercial UV-vis spectrometers.
