# Supplementary material for: Microsecond time-scale kinetics of transient biochemical reactions
Source: PLoS One. 2017 Oct 3;12(10):e0185888. doi: 10.1371/journal.pone.0185888 (PMC5626514; doi:10.1371/journal.pone.0185888)
Supplement: S2 Fig — Absorbance spectra of ferro- and ferricytochrome c without fluid flow (blue) and with a fluid flow (red) rate of 20 mL min-1. Bottom spectra are ferricytochrome c. The spectra of ferrocytochrome c are offset by +0.5 for clarity. The spectra are the average of the spectra recorded in pixels 0–1900, along the whole length of the flow-cell. (PDF) [file pone.0185888.s005.pdf]

S2 Fig. Comparison between static absorbance spectra and spectra recorded during fluid flow.

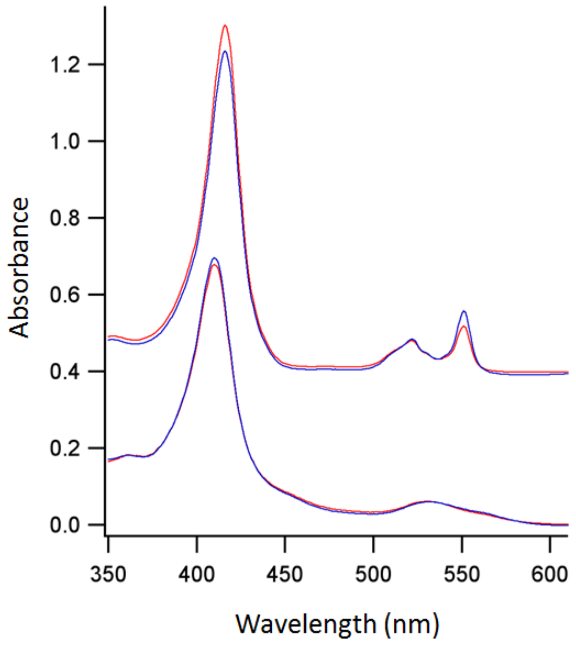

Absorbance spectra of ferro- and ferricytochrome *c* without fluid flow (blue) and with a fluid flow (red) rate of 20 mL min^{-1}. Bottom spectra are ferricytochrome *c*. The spectra of ferrocytochrome *c* are offset by +0.5 for clarity. The spectra are the average of the spectra recorded in pixels 0 – 1900, along the whole length of the flow-cell.
