# Supplementary material for: Microsecond time-scale kinetics of transient biochemical reactions
Source: PLoS One. 2017 Oct 3;12(10):e0185888. doi: 10.1371/journal.pone.0185888 (PMC5626514; doi:10.1371/journal.pone.0185888)
Supplement: S3 Fig — (a) The reference absorbance spectra: acid control spectra (black: 3 mM HPTS in 10 mM HCl (pH 2) mixed vs 10 mM HCl) and base control spectra (blue: 3mM HPTS in 10 mM NaOH (pH 12) mixed vs 10 mM NaOH) recorded within the first 7 μs (20 pixels) after dead time at a liquid flow rate of 20 mL min-1. (b) Absorbance spectra of the reaction of 3 mM HPTS in 20 mM HCl (pH 1.7) mixed with 30 mM NaOH (pH 12.48) recorded within the first 7 μs (20 pixels) after dead time at a liquid flow rate of 20 mL min-1. (c) Kinetic traces of the reactions determined in the first 64 pixels (22.4 μs) after dead time at a liquid flow rate of 20 mL min-1. The spectra were analysed using two-component analysis. Their normalized intensities are found to be close to zero and one, respectively, from the first data point onwards, indicating complete conversion from the acid to base form of HPTS. (d) Percentage of mixing in the first time point of the rectangular cuvette as a function of the flow rate and the Reynolds number calculated for the 100 μm Pt inlay orifice. Percentage of mixing is presented as appearance of base (blue) and disappearance of acid (black). (PDF) [file pone.0185888.s006.pdf]

S3 Fig. HPTS acid to base reaction.

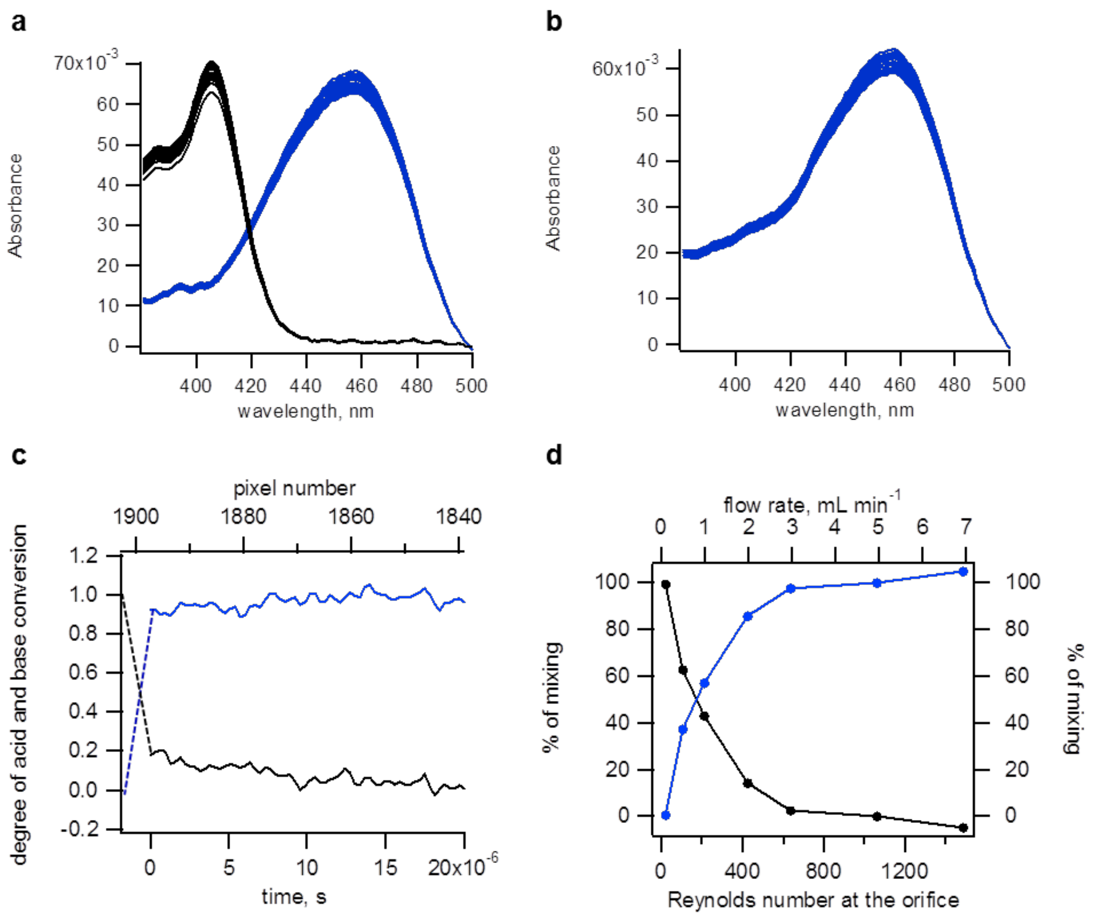

(a) The reference absorbance spectra: acid control spectra (black: 3 mM HPTS in 10 mM HCl (pH 2) mixed vs 10 mM HCl) and base control spectra (blue: 3mM HPTS in 10 mM NaOH (pH 12) mixed vs 10 mM NaOH) recorded within the first 7 µs (20 pixels) after dead time at a liquid flow rate of 20 mL min^{-1}. (b) Absorbance spectra of the reaction of 3 mM HPTS in 20 mM HCl (pH 1.7) mixed with 30 mM NaOH (pH 12.48) recorded within the first 7 µs (20 pixels) after dead time at a liquid flow rate of 20 mL min^{-1}. (c) Kinetic traces of the reactions determined in the first 64 pixels (22.4 µs) after dead time at a liquid flow rate of 20 mL min^{-1}. The spectra were analysed using two-component analysis. Their normalized intensities are found to be close to zero and one, respectively, from the first data point onwards, indicating complete conversion from the acid to base form of HPTS. (d) Percentage of mixing in the first time point of the rectangular cuvette as a function of the flow rate and the Reynolds number calculated for the 100 µm Pt inlay orifice. Percentage of mixing is presented as appearance of base (blue) and disappearance of acid (black).
