# Supplementary material for: Microsecond time-scale kinetics of transient biochemical reactions
Source: PLoS One. 2017 Oct 3;12(10):e0185888. doi: 10.1371/journal.pone.0185888 (PMC5626514; doi:10.1371/journal.pone.0185888)
Supplement: S4 Fig — Ferrocytochrome c was 29.6 μM. Sodium ferrihexacyanide concentrations were 0.001 (red), 0.002, 0.003, 0.006, 0.01 and 0.02 (black) mM. The buffer was 50 mM potassium phosphate, pH 7.0. The black solid lines are second-order fits used to determine the rate constant of the reaction. The fraction of reduced cytochrome c has been plotted (data points) as calculated with two-component analysis from the absorption at 550 nm at a temperature of (a) 19.3°C and (b) 34.4°C. (PDF) [file pone.0185888.s007.pdf]

S4 Fig. Stopped-flow kinetic traces of the oxidation of ferrocytochrome *c* by various concentrations of sodium ferrihexacyanide.

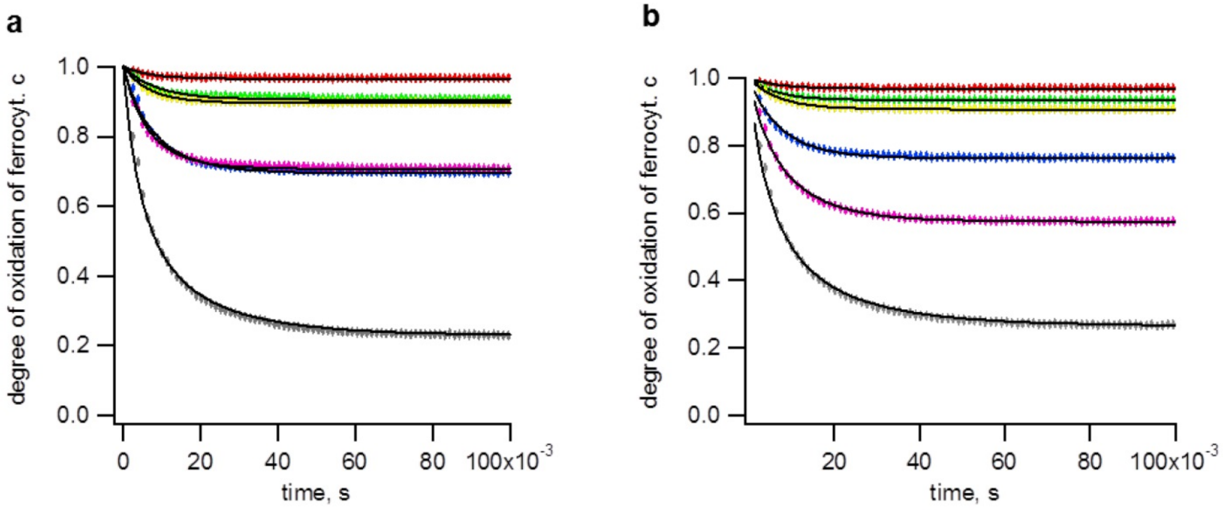

Ferrocytochrome *c* was 29.6 μM. Sodium ferrihexacyanide concentrations were 0.001 (red), 0.002, 0.003, 0.006, 0.01 and 0.02 (black) mM. The buffer was 50 mM potassium phosphate, pH 7.0. The black solid lines are second-order fits used to determine the rate constant of the reaction. The fraction of reduced cytochrome *c* has been plotted (data points) as calculated with two-component analysis from the absorption at 550 nm at a temperature of (a) 19.3^{o}C and (b) 34.4^{o}C.
