# Supplementary material for: Microsecond time-scale kinetics of transient biochemical reactions
Source: PLoS One. 2017 Oct 3;12(10):e0185888. doi: 10.1371/journal.pone.0185888 (PMC5626514; doi:10.1371/journal.pone.0185888)
Supplement: S5 Fig — 0.5 mM ferrocytochrome c and 20 mM sodium ferrihexcyanide were in 50 mM potassium phosphate buffer, pH 7.0. (a) Absorbance spectra recorded in the time span from 3.8 μs to 70 μs after initiation of the reaction. The spectrum with the largest amplitude (blue) at 415 and 550 nm represents the t = 0 sample, recorded separately. The figure shows 190 spectra, i.e. the time resolution of the spectra is 350 ns. (b) Selection of spectra of ferrocytochrome c in reaction with ferrihexacyanide (red) and comparison to the spectra of ferri- (black) and ferrocytochrome c (blue) that served as reference spectra to calculate the amount of conversion by component analysis. (PDF) [file pone.0185888.s008.pdf]

S5 Fig. Absorbance spectra of oxidation of cytochrome *c* by sodium ferrihexacyanide.

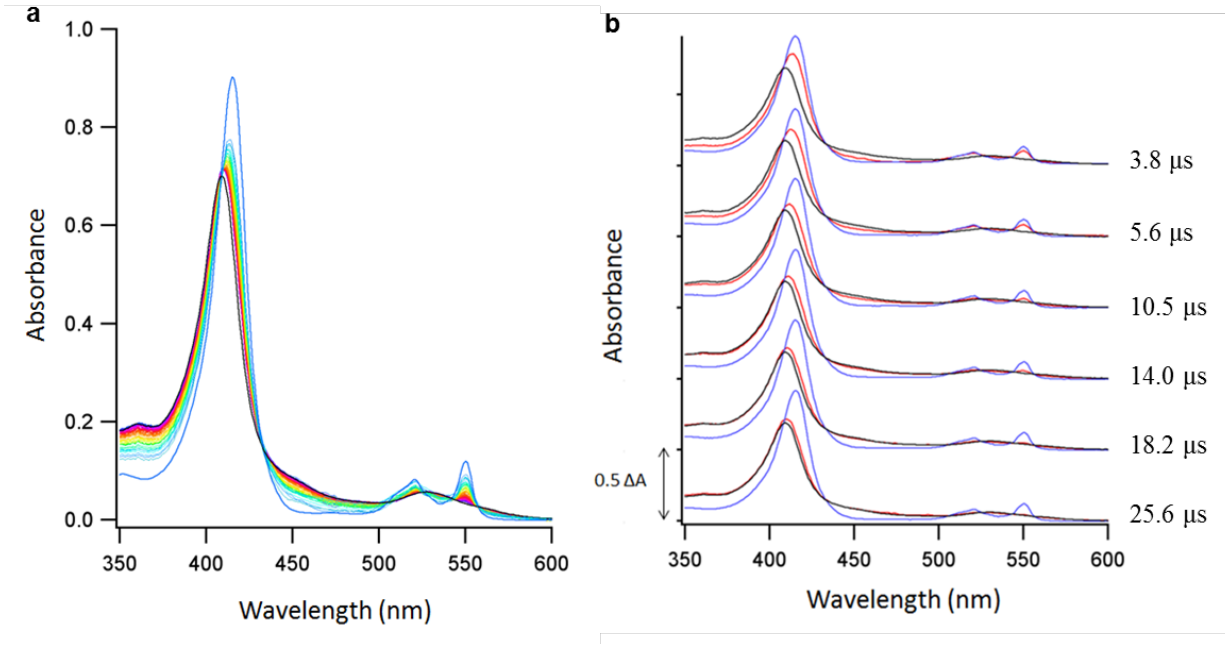

0.5 mM ferrocytochrome c and 20 mM sodium ferrihexcyanide were in 50 mM potassium phosphate buffer, pH 7.0. (a) Absorbance spectra recorded in the time span from 3.8 µs to 70 µs after initiation of the reaction. The spectrum with the largest amplitude (blue) at 415 and 550 nm represents the t=0 sample, recorded separately. The figure shows 190 spectra, i.e. the time resolution of the spectra is 350 ns. (b) Selection of spectra of ferrocytochrome *c* in reaction with ferrihexacyanide (red) and comparison to the spectra of ferri- (black) and ferrocytochrome *c* (blue) that served as reference spectra to calculate the amount of conversion by component analysis.
