# Supplementary material for: Microsecond time-scale kinetics of transient biochemical reactions
Source: PLoS One. 2017 Oct 3;12(10):e0185888. doi: 10.1371/journal.pone.0185888 (PMC5626514; doi:10.1371/journal.pone.0185888)
Supplement: S1 Table — (PDF) [file pone.0185888.s009.pdf]

S1 Table. Rate constants at 0.1 M ionic strength for the reaction between ferrocytochrome *c* and sodium ferrihexacyanide.

Analysis Method	Rate constant (M^{-1} s^{-1})
**Stopped-flow**	
550 nm peak	
19.3^{o}C	(0.88 ± 0.23) x 10^{7}
34.4^{o}C	(0.94 ± 0.1) x 10^{7}
**Two-component analysis**	
19.3^{o}C	(1.03 ± 0.14) x 10^{7}
34.4^{o}C	(1.05 ± 0.07) x 10^{7}
**Continuous-flow set up**	
27.5^{o}C	(0.96 ± 0.03) x 10^{7}
